# The College of Podiatry Annual Conference 2015: meeting abstracts: Harrogate, Yorkshire, UK. 19–21 November 2015

**DOI:** 10.1186/s13047-016-0141-x

**Published:** 2016-04-27

**Authors:** Jasper W. K. Tong, Veni P. Kong, Lily Sze, Susie Gale, John Veto, Carla McArdle, Thanaporn Tunprasert, Victoria Bradley, Siobhan Strike, Thanaporn Tunprasert, Victoria Bradley, Siobhan Strike, Robert Ashford, Roozbeth Naemi, Nachiappan Chocklingam, Xavi de Blasc, Lisa Farndon, Vicki Robinson, Emily Nicholls, Tabitha Birch, Ivan Birch, Simon Otter, Sunil Kumar, Peter Gow, Nicola Dalbeth, Michael Corkill, Kevin Davies, Sam Panthakalam, Maheswaran Rohan, Keith Rome, Chloe Egan, Lisa Chandler, Peta Tehan, Vivienne Chuter, Jennifer Sonter, Sean Lanting, Lorna Hicks, Christopher Joyce, David Watterson, Caroline McIntosh, Christopher Joyce, Nigel Roberts, Jacqueline Forss, Chrystalla Charalambous, Jack Kirby, Oluwakemi Ojo, Jacqueline Forss, Sarah Caukill, Jacqueline Capon, Radiance Fong, Louis Loy, Matthew Diment, Madeleine Murray, Mairghread Ellis, Carla McArdle, Peta Tehan, Vivienne Chuter, Christopher Oldmeadow, Nicola Carey, Karen Stenner, Heather Gage, Jane Brown, Peter Williams, Simon Otter, Ann Moore, Jude Edwards, Freda Mold, Molly Courtenay, Peta Tehan, Alan Bray, Vivienne Chuter, Pamela Hindmoor, Craig Gwynne, Sarah Curran, Andy Bridgen, Caroline Fairhurst, Joy Adamson, Belen Corbacho Martin, Sarah Cockayne, Catherine Hewitt, Kate Hicks, Anne-Maree Keenan, Lorraine Loughrey-Green, Hylton Menz, Anthony Redmond, Sara Rodgers, Jude Watson, David Torgerson, Robin Hull, Sarah Lamb, Caroline McIntosh, Wesley Vernon, Lisa Farndon, Lorraine Loughrey-Green, Sarah Cockayne, Anthony Redmond, Anne-Maree Keenan, Sara Rodgers, Lisa Farndon, Wesley Vernon, David Torgerson, Caroline Fairhurst, Jude Watson, Hylton Menz, Sarah Lamb, Robin Hull, Gavin Wylie, Zoe Young, Brian Williams, Frank Sullivan, Hylton Menz, Simon Ogston, Jacqui Morris, Cathy Bowen, Dorit Kunkel, Mark Cole, Margaret Donovan-Hall, Ruth Pickering, Malcolm Burnett, Dan Bader, Judy Robison, Louis Mamode, Ann Ashburn, Peter McQueen, Maxine Daniels, Michael Doherty, Nigel Arden, Cathy Bowen, Charlotte Dando, Lindsey Cherry, Cathy Bowen, Nichola Stefanou, Sarah Cockayne, Joy Adamson, Caroline Fairhurst, Catherine Hewitt, Anne-Maree Keenan, Sally Lamb, Lorraine Loughrey-Green, Caroline McIntosh, Hylton Menz, Anthony Redmond, Sara Rodgers, Wesley Vernon, Jude Watson, Lisa Farndon, Belen Corbacho, Robin Hull, David Torgerson, Begonya Alcacer-Pitarch, Anthony Redmond, Maya Buch, Anne-Maree Keenan

**Affiliations:** Allied Health Specialties, KK Women’s and Children’s Hospital, Singapore, Singapore; Physical Education and Sports Science Academic Group, National Institute of Education, Nanyang Technological University, Singapore, Singapore; Queen Margaret University, Musselburgh, Scotland; University of Brighton, Brighton, UK; Ministry of Defence, Headley Court Complex Trauma Department, Surrey, UK; University of Roehampton, London, UK; University of Brighton, Brighton, UK; Ministry of Defence, Headley Court Complex Trauma Department, Surrey, UK; University of Roehampton, London, UK; Birmingham City University, Birmingham, UK; Staffordshire University, Stoke-on-Trent, UK; Universitat Ramon Llun, Barcelona, Spain; Sheffield Teaching Hospitals NHS Foundation Trust, Sheffield, UK; Sociological Studies, University of Sheffield, Sheffield, UK; University of Roehampton, London, UK; Sheffield Teaching Hospitals NHS Foundation Trust, Sheffield, UK; University of Brighton, Brighton, UK; Counties Manuaku District Health Board, Auckland, New Zealand; Auckland District Health Board, Auckland, New Zealand; Waitemata District Health Board, Auckland, New Zealand; Brighton & Sussex Medical School, Brighton, UK; East Sussex HealthcareTrust, Eastbourne, UK; AUT University, Auckland, New Zealand; Cambridgeshire and Peterborough NHS Foundation Trust, Cambridge, UK; University of Northampton, Northampton, UK; University of Newcastle, Newcastle upon Tyne, UK; Betsi Cadwaladr University Health Board, Bangor, Wales UK; National University of Ireland, Galway, Ireland; Health Service Executive West, Galway, Ireland; National University of Ireland, Galway, Galway, Ireland; University of Brighton, Brighton, UK; University of Brighton, Brighton, UK; Queen Margaret University, Musselburgh, Scotland; University of Newcastle, Newcastle upon Tyne, Tyne and Wear UK; Hunter Medical Research Institute, 1 Kookaburra Circuit, New Lambton Heights, NSW Australia; University of Brighton, Lewes Rd, Brighton, UK; University Of Surrey, Guildford, UK; Liverpool John Moore University, 70 Mount Pleasant, Liverpool, UK; Cardiff University, Cardiff, UK; University of Newcastle, Newcastle upon Tyne, Tyne and Wear UK; Vascular Health Care, East Maitland, NSW Australia; New College Durham, Framwellgate Moor Campus, Framwellgate, Durham, UK; Cardiff Metropolitan University, Western Ave, Cardiff, UK; University of Huddersfield, Queensgate, Huddersfield UK; York Trials Unit, University of York, Heslington, York UK; NIHR Leeds Musculoskeletal Biomedical Research Unit, Chapel Allerton Hospital, Leeds, UK; Leeds Institute of Rheumatology and Musculoskeletal Medicine, University of Leeds, Leeds, UK; Faculty of Health Sciences, Lower Extremity and Gait Studies Program, La Trobe University, Bundoora, Victoria Australia; Podiatry Services, Harrogate and District NHS Foundation Trust, Harrogate District Hospital, Lancaster Park Rd, Harrogate, UK; Nuffield Department of Orthopaedics, Rheumatology and Musculoskeletal Sciences, Kadoorie Critical Care Research Centre, John Radcliffe Hospital, University of Oxford, Headley Way, Oxford, UK; National University of Ireland, University Rd, Galway, Ireland; Podiatry Services, Sheffield Teaching Hospitals NHS Foundation Trust, Jordanthorpe Health Centre, 1 Dyche Close, Sheffield, UK; Podiatry Services, Sheffield Primary Care Trust, Fulwood House, Old Fulwood Road, Sheffield, UK; Leeds Institute of Rheumatic and Musculoskeletal Medicine and NIHR Leeds Musculoskeletal Biomedical Research Unit, Leeds, UK; York Trials Unit, Department of Health Sciences, University of York, York, UK; Podiatry Services, Sheffield PCT, Jordanthorpe Health Centre, Sheffield, UK; La Trobe University, Melbourne, Australia; Nuffield Department of Orthopaedics Rheumatology and Musculoskeletal Sciences, University of Oxford, Oxford, UK; Warwick Clinical Trials Unit, University of Warwick, Coventry, UK; Podiatry Services, Harrogate and District NHS Foundation Trust, Harrogate District Hospital, Harrogate, UK; School of Nursing and Midwifery, University of Dundee, Dundee, Scotland; Department of Podiatry, Allied Health Professions Directorate, NHS Tayside, Dundee, Scotland; Nursing Midwifery and Allied Health Professions Research Unit, Stirling University, Stirling, Scotland; Department of Family & Community Medicine, University of Toronto, North York General Hospital, Toronto, Canada; Lower Extremity and Gait Studies Program, Melbourne, VIC Australia; Centre for Biomedical Science and Public Health, University of Dundee, Dundee, Scotland; Faculty of Health Sciences, University of Southampton, Southampton, UK; Faculty of Medicine, University of Southampton, Southampton, UK; University of Southampton, Southampton, UK; University of Oxford, Oxford, UK; Barts Health NHS Trust, London, UK; University of Nottingham, Nottingham, UK; Medical Research Centre, Southampton, UK; University of Southampton, Southampton, UK; Solent NHS Trust, Southampton, UK; Sunderland University, Sunderland, UK; Department of Health Sciences, Faculty of Sciences, University of York, York, UK; NIHR Leeds Musculoskeletal Biomedical Research Unit, Leeds, UK; Leeds Institute of Rheumatic and Musculoskeletal Medicine, Leeds, UK; Nuffield Department of Orthopaedics, Rheumatology and Musculoskeletal Sciences, Kadoorie Critical Care Research Centre, John Radcliffe Hospital, University of Oxford, Oxford, UK; School of Health Sciences, Áras Moyola, NUI Galway, Galway, Ireland; School of Allied Health, College of Science, Health and Engineering, La Trobe University, Victoria, Australia; Podiatry Services, Sheffield Teaching Hospitals NHS Foundation Trust, Jordanthorpe Health Centre, Sheffield, UK; Podiatry Services, Harrogate and District NHS Foundation Trust, Monkgate Health Centre, York, UK; Leeds Institute of Rheumatic and Musculoskeletal Medicine, Leeds, UK; Leeds NIHR Biomedical Research Unit, Leeds, UK

## Abstract

P3 Medial longitudinal arch development of school children

Jasper W.K. Tong, Veni P. Kong

P4 Is measuring the subtalar joint reliable?

Lily Sze, Susie Gale, John Veto, Carla McArdle

P5 Comparison of turning gait biomechanics between able-bodied and unilateral transtibial amputee participants

Thanaporn Tunprasert, Victoria Bradley, Siobhan Strike

P6 Comparison of walking gait biomechanics between able-bodied and unilateral transtibial amputee participants using a new model of energy-storage-and-return (ESAR) prosthetic

Thanaporn Tunprasert, Victoria Bradley, Siobhan Strike

P7 An observational study of in-shoe plantar and dorsal pressures of skilled downhill skiers on a dry ski slope

Robert Ashford, Roozbeth Naemi, Nachiappan Chocklingam, Xavi de Blasc

P8 If the shoe fits: a footwear choice toolkit informed by social science methodologies

Lisa Farndon, Vicki Robinson, Emily Nicholls

P9 The identification of emotions from gait

Tabitha Birch, Ivan Birch

P11 Experience of foot problems in patients with systemic lupus erythematosus

Simon Otter, Sunil Kumar, Peter Gow, Nicola Dalbeth, Michael Corkill, Kevin Davies, Sam Panthakalam, Maheswaran Rohan, Keith Rome

P14 Negative pressure wound therapy for the management of foot wounds in the diabetic population: a review of the literature

Chloe Egan, Lisa Chandler

P15 Lower limb vascular assessment in diabetes: a multifaceted assessment of objective screening techniques

Peta Tehan, Vivienne Chuter, Jennifer Sonter, Sean Lanting

P16 Improving outcomes for diabetes foot complications

Lorna Hicks

P17 Acupuncture… an alternative or adjunctive treatment option for diabetes-related neuropathic pain?

Christopher Joyce, David Watterson, Caroline McIntosh

P18 “My back is in agony” – A cross-sectional study into the relationship between musculoskeletal complaints and a whole body postural risk assessment in podiatry students

Christopher Joyce, Nigel Roberts

P19 Swabs of the treatment couches: Does the material type and texture of podiatric treatment couches increase microorganism contamination?

Jacqueline Forss, Chrystalla Charalambous, Jack Kirby, Oluwakemi Ojo

P20 Does increased exudate viscosity effect the absorption rate of exudate into four different wound dressings?

Jacqueline Forss, Sarah Caukill, Jacqueline Capon, Radiance Fong, Louis Loy

P21 An investigation into the microbial load of a 40 °C and 60 °C wash

Matthew Diment, Madeleine Murray, Mairghread Ellis, Carla McArdle

P23 The sensitivity and specificity of the toe brachial index in detecting peripheral arterial disease: a systematic review and meta-analysis

Peta Tehan, Vivienne Chuter, Christopher Oldmeadow

P24 Medicines management activities and non-medical prescribing within podiatry and physiotherapy: an integrative review of the literature

Nicola Carey, Karen Stenner^,^ Heather Gage, Jane Brown, Peter Williams, Simon Otter, Ann Moore, Jude Edwards, Freda Mold, Molly Courtenay

A7.2 Non-invasive vascular assessment in the foot with Diabetes: Diagnostic accuracy of ankle brachial index, toe brachial index and continuous wave Doppler

Peta Tehan, Alan Bray, Vivienne Chuter

A7.5 The efficacy of dressings on post nail surgery phenolised wounds

Pamela Hindmoor

B7.1 Cross-sectional study investigating the role of proximal and distal factors in the development of patellofemoral joint pain

Craig Gwynne, Sarah Curran

B7.2 Podiatrist’s interpretation and use of evidence in MSK practice

Andy Bridgen

B7.4 Predictors of falling in older podiatry patients – findings from the REFORM study

Caroline Fairhurst, Dr Joy Adamson, Belen Corbacho Martin, Sarah Cockayne, Prof Catherine Hewitt, Kate Hicks, Anne-Maree Keenan, Lorraine Loughrey-Green, Hylton Menz, Anthony Redmond, Sara Rodgers, Jude Watson, David Torgerson, Robin Hull, Sarah Lamb, Caroline McIntosh, Wesley Vernon, Lisa Farndon

B7.5 The REFORM study: Insole preference, requirements and compliance of podiatry patient’s aged 65 and over and at risk of falling

Lorraine Loughrey-Green, Sarah Cockayne, Anthony Redmond, Anne-Maree Keenan, Sara Rodgers, Lisa Farndon, Wesley Vernon, David Torgerson, Caroline Fairhurst, Jude Watson, Hylton Menz, Sarah Lamb, Robin Hull

B7.6 A podiatry intervention to reduce falls in care home residents is feasible and demonstrates benefits: results from PIRFECT, a feasibility randomised controlled trial

Gavin Wylie, Zoe Young, Brian Williams, Frank Sullivan, Hylton Menz, Simon Ogston, Jacqui Morris

C7.1 A survey exploring footwear habits in people with stroke and people with Parkinson’s

Cathy Bowen, Dorit Kunkel, Mark Cole, Margaret Donovan-Hall, Ruth Pickering, Malcolm Burnett, Dan Bader, Judy Robison, Louis Mamode, Ann Ashburn

C7.2 Painful foot osteoarthritis; a common symptom in a common pathology?

Peter McQueen, Maxine Daniels, Michael Doherty, Nigel Arden, Cathy Bowen

C7.4 Clinical diagnosis of symptomatic forefoot neuroma in the general population: Delphi based recommendations

Charlotte Dando, Lindsey Cherry, Cathy Bowen

C7.5 The development and implementation of a Clinical Quality Improvement Framework suitable for use in community services

Nichola Stefanou

C7.6 The REFORM study - methodological considerations in running a cohort randomised controlled trial within a podiatry patient caseload

Sarah Cockayne, Joy Adamson, Caroline Fairhurst, Catherine Hewitt, Anne-Maree Keenan, Sally Lamb, Lorraine Loughrey-Green, Caroline McIntosh, Hylton Menz, Anthony Redmond, Sara Rodgers, Wesley Vernon, Jude Watson, Lisa Farndon, Belen Corbacho, Robin Hull, David Torgerson

A31 Jewel in the crown: Exploring the factors contributing to the development and impact of foot problems in Systemic Sclerosis (SSc)

Begonya Alcacer-Pitarch, Anthony Redmond, Maya Buch, Anne-Maree Keenan

## P3 Medial longitudinal arch development of school children

### Jasper W.K. Tong^1^, Veni P. Kong^2^

#### ^1^Allied Health Specialties, KK Women’s and Children’s Hospital, Singapore; ^2^Physical Education and Sports Science Academic Group, National Institute of Education, Nanyang Technological University, Singapore

##### **Correspondence:** Jasper W.K. Tong - Allied Health Specialties, KK Women’s and Children’s Hospital, Singapore

**Background**

Foot structure is often classified into flat foot, neutral and high arch type based on the variability of the Medial Longitudinal Arch (MLA). To date, the literature provided contrasting evidence on the age when MLA development stabilises in children. The influence of footwear on MLA development is also unknown.

**Aim**

This study aims to (i) clarify whether the MLA is still changing in children from age 7 to 9 years old and (ii) explore the relationship between footwear usage and MLA development, using a longitudinal approach.

**Methods**

We evaluated the MLA of 111 healthy school children [age = 6.9 (0.3) years] using three parameters [arch index (AI), midfoot peak pressure (PP) and maximum force (MF: % of body weight)] extracted from dynamic foot loading measurements at baseline, 10-month and 22-month follow-up. Information on the type of footwear worn was collected using survey question. Linear mixed modelling was used to test for differences in the MLA over time.

**Results**

Insignificant changes in all MLA parameters were observed over time [AI: P = .15; PP: P = .84; MF: P = .91]. When gender was considered, the AI of boys decreased with age [P = .02]. Boys also displayed a flatter MLA than girls at age 6.9 years [AI: mean difference = 0.02 (0.01, 0.04); P = .02]. At baseline, subjects who wore close-toe shoes displayed the lowest MLA overall [AI/PP/MF: P < .05]. Subjects who used slippers when commencing footwear use experienced higher PP than those who wore sandals [mean difference = 31.60 (1.44, 61.75) kPa; post-hoc P = .04].

**Discussion and conclusion**

Our findings suggested that the MLA of children remained stable from 7 to 9 years old, while gender and the type of footwear worn during childhood may influence MLA development. Clinicians may choose to commence therapy when a child presents with painful flexible flat foot at age 7 years, and may discourage younger children from wearing slippers when they commence using footwear.

## P4 Is measuring the subtalar joint reliable?

### Lily Sze, Susie Gale, John Veto, Carla McArdle

#### Queen Margaret University, Musselburgh, Scotland

##### **Correspondence:** Lily Sze - Queen Margaret University, Musselburgh, Scotland

**Background**

Biomechanical assessments of the rearfoot in static closed kinetic chain are routinely performed in podiatry clinics to help with the diagnosis of foot pathologies/lower limb disorders and typically are used to inform orthotic prescriptions. However existing literature reports inter-rater and intra-rater reliabilities of such measurements are generally poor. (Menz 1995; Menz and Keenan 1997; Payne and Richardson 2000; Robinson et al. 2001; Keenan and Bach 2006; Chevillotte et al. 2009; Jarvis et al. 2012; Schepper et al. 2012).

Factors that affect measurement reliability include the determination of calcaneal bisection lines and identification of the subtalar joint (STJ) neutral position.

**Aim**

The study aimed to evaluate the inter-rater and intra-rater reliability of RCSP and NCSP in order to add to the current evidence relating to the reliability of biomechanical assessments within a podiatric context.

**Methods**

Following a consent process 30 volunteer podiatry students (excluding first year podiatry students) measured the RCSP and NCSP of two subjects (A and B) in static closed kinetic chain. Subject A had a pre-bisected calcaneus prior to participants measuring calcaneal positions. Subject B in comparison required each participant to bisect the calcanei prior to measuring calcaneal positions. All participants returned 7 days later to repeat the experimental protocol.

Inter- rater reliability was determined by analysing variations in measurements of NCSP and RCSP for subjects A and B among participants. Intra- rater reliability was determined by analysing measurements of RCSP and NCSP of each individual participant between session 1 and session 2. In order to obtain reliability of results the same type of protractor was used to conduct all measurements.

**Results**

Intra–class correlation coefficient (ICC) values adapted from Dancey and Reidy (2004) were used to determine levels of similarity of correlation between measurements.

Using this scale inter-rater reliability for subject A and B showed zero/weak correlation.

Intra-rater reliability between measurements of each individual participant was moderate to strong for 90 % of participants.

**Discussion and conclusion**

The study supports existing literature in questioning the use of unreliable biomechanical measurements within clinical practice, potentially jeopardizing the provision of safe and effective clinical care.

**Ethical approval statement**

Ethical approval was granted from the university divisional research ethics committee.

**Acknowledgements**

Recognition is given to members of the research team Christopher Kelly, Corrine Dickson, Fal Osman, Kerry Moffat and Laura Bew.

## P5 Comparison of turning gait biomechanics between able-bodied and unilateral transtibial amputee participants

### Thanaporn Tunprasert^1^, Victoria Bradley^2^, Siobhan Strike^3^

#### ^1^University of Brighton, Brighton, UK; ^2^Ministry of Defence, Headley Court Complex Trauma Department, Surrey, UK; ^3^University of Roehampton, London, UK

##### **Correspondence:** Thanaporn Tunprasert - University of Brighton, Brighton, UK

**Aim**

The aim of this study is to compare the biomechanics of turning movement between the able-bodied and unilateral transtibial amputee populations.

**Background**

The ability to change direction during walking is essential for a person to maintain their mobility level. In daily activity, turning movement can be accounted for 20 % of the steps taken to complete an outdoor trip and up to 50 % for an indoor ambulation. Turning movement is a complex reorientation mechanism of the body which can lead to postural instability. Specific compensatory mechanisms may also be required in populations with abnormal gait (e.g. amputees, subjects with hip arthritis) in order to successfully complete a turn. One example is the use of step turning strategy rather than spin turning strategy as the former strategy provides a better stability during ambulation.

**Methods**

Seven unilateral transtibial amputees and nine able-bodied participants were recruited for this study. The amputees used the same model of energy-storage-and-return (ESAR) prosthetic. A nine-camera Vicon system with a sampling rate of 100Hz synchronized with Kistler force platforms with a sampling rate of 1000Hz were used to record the spatio-temporal (S-T) and kinetic data. Participants were asked to walk at their own comfortable walking speed along the dashed lines marked on the floor indicating the walking path and the 90° turning point. The two populations were subdivided into three limb types: able-bodied limb (of able-bodied participants), intact limb (of amputee participants), and prosthetic limb (of amputee participants).

**Results**

In the able-bodied participants, step- and spin- turn strategies were employed almost equally. However, the amputee participants used mainly the step-turn strategy and much less the spin-turn strategy. Thus, only the step-turn trials were further analysed in this study to allow better comparison of other biomechanical parameters of step-turning movement. The participants exhibited a significantly slower speed for the turning gait (1.08 to 1.14 m/s) compared to walking gait without a turn (1.37 to 1.49 m/s; p < 0.05). The approach step length (before a turn) was shorter compared to the turning step length (during a turn) in the able-bodied and intact limbs but remained comparable in the prosthetic limb. Significant differences were found in some peak values of the joint powers, particularly in the ankle, knee and hip in the sagittal plane, and the hip in the frontal plane (p < 0.05). For example, a significantly higher absorption peak at the ankle joint in the sagittal plane (A1S) was found in the prosthetic limb (0.63 W/kg) compared to the able-bodied (0.22 W/kg) and intact (0.16 W/kg) limbs.

**Discussion and conclusion**

To achieve a step-turning movement, the amputee participants required unique compensatory mechanisms which affected not only the prosthetic limb but also the intact limb. These mechanisms were also different from the methods employed by the able-bodied subjects.

## P6 Comparison of walking gait biomechanics between able-bodied and unilateral transtibial amputee participants using a new model of energy-storage-and-return (ESAR) prosthetic

### Thanaporn Tunprasert^1^, Victoria Bradley^2^, Siobhan Strike^3^

#### ^1^University of Brighton, Brighton, UK; ^2^Ministry of Defence, Headley Court Complex Trauma Department, Surrey, UK; ^3^University of Roehampton, London, UK

##### **Correspondence:** Thanaporn Tunprasert - University of Brighton, Brighton, UK

**Aim**

The aim of this study is to compare the biomechanics of straight-line walking movement between the able-bodied and unilateral transtibial amputee populations using a new model of energy-storage-and-return (ESAR) prosthetic.

**Background**

Traditional passive prosthetic feet provided limited ankle power output resulting in an increase in hip joint work in the ipsilateral limb and/or compensatory mechanisms in the contralateral limb without amputation. The energy-storage-and-return (ESAR) prosthetics were found to generally provide better ankle power absorption and generation compared to stiff ankle prosthetics.

**Methods**

Seven unilateral transtibial amputees and nine able-bodied participants were recruited for this study. The amputees used the same blade-style model of energy-storage-and-return (ESAR) prosthetic. A nine-camera Vicon system with a sampling rate of 100Hz synchronized with Kistler force platforms with a sampling rate of 1000Hz were used to record the spatio-temporal (S-T) and kinetic data. Participants were asked to walk at their own comfortable walking speed along the 7.5 m walkway with the force platforms located approximately in the middle. The two populations were subdivided into three limb types: able-bodied limb (of able-bodied participants), intact limb (of amputee participants), and prosthetic limb (of amputee participants).

**Results**

The participants chose to walk at a similar walking speed (1.37 to 1.49 m/s). The average result of the maximal blade deformation during stance was 8.21 mm. The significant difference is set at p < 0.05. The prosthetic limb was found to have the lowest ground reaction forces (GRFs) at the propulsion peak of vertical GRF (9.68 N/kg) and the lateral peak of medial-lateral GRF (0.07 N/kg) in comparison to the able-bodied (11.00 and 0.32 N/kg) and intact (11.42 and 0.39 N/kg) limbs. The intact limb also exhibited the highest braking force (2.30 N/kg) compared to the able-bodied (1.81 N/kg) and prosthetic (1.38 N/kg) limbs. The prosthetic limb showed the highest joint absorption powers at the ankle joint in the sagittal plane (1.29 W/kg) and at the ankle and knee joint in the frontal plane (0.12 and 0.80 W/kg). Similarly, the prosthetic limb demonstrated the highest joint generation power at the hip joint in the sagittal plane (1.11 W/kg) compared to the able-bodied (0.45 W/kg) and intact (0.19 W/kg) limbs.

**Discussion and conclusion**

The amputees using the new blade-style ESAR prosthetic exhibited the higher joint absorption powers at the ankle and knee and the higher joint generation power at the hip in the prosthetic limb. However, possible compensatory mechanisms were also displayed in the intact limb (e.g. the higher braking force). Although the new model of ESAR prosthetics provided a better performance compared to stiff ankle prosthetics, the results were still not all comparable to the results from the able-bodied participants. Further studies are suggested to identify specific mechanical properties of the ESAR prosthetic in association with the biomechanical results of the amputees to increase the understanding of the compensatory mechanisms in the unilateral transtibial amputee subjects.

## P7 An observational study of in-shoe plantar and dorsal pressures of skilled downhill skiers on a dry ski slope

### Robert Ashford^1,2^, Roozbeth Naemi^2^, Nachiappan Chocklingam^2^, Xavi de Blasc^3^

#### ^1^Birmingham City University, Birmingham, UK; ^2^Staffordshire University, Stoke-on-Trent, UK; ^3^Universitat Ramon Llun, Barcelona, Spain

##### **Correspondence:** Robert Ashford - Birmingham City University, Birmingham, UK

**Background**

Downhill skiing is a very popular winter sport. There has been a plethora of research into many aspects of skiing including: skiing techniques, ski injuries; ski materials – boots, skiwear and ski equipment. There has also been research into pressure distribution on the sole in ski boots and pressure distribution on the ski but there has been very little research in correlating pressure on the plantar aspect of the ski boot during skiing and the pressure associated with straight downhill skiing and the ski turn. In addition the symmetry of the turn has not been fully researched.

**Aim**

The aim of this study was to investigate pressure data from the anterior aspect of the lower limb and the sole of the foot whilst the skier undertook specific skiing manoeuvres.

**Methods**

Ethical approval was sought and granted from the University Ethics Committee. A single case methodology was adopted. One experienced male skier was recruited: aged 32; was a ski instructor; classified as a slalom skier; had 16 years skiing experience The skier was instructed to follow a predetermined ski path as outlined by markers on the ski slope and make appropriate turns when the markers veered either left or right. Markers were placed at angles of 200, 400 and 600 from the mid-line. However to facilitate a smooth turn the corner of the angle was curved slightly. Each ski boot was fitted with sensors (Kinematrix, Portugal) attached to: hallux, 1and 5 met heads; central plantar heel; bony apex of the medial and lateral malleolus, medial and lateral of the tibia at position of ½ the shank length. A total of three runs per manoeuvre were recorded. When person turned left, left foot was considered 'inside foot' and right foot was considered 'outside foot'. This method was also adopted when a right turn was initiated: right foot was considered 'inside foot' and left foot was considered 'outside foot'. A boolean, T-test and Wilcoxon tests were used to compare pressure on each sensor on the inside/outside of foot. ANOVA and Kruskal-Wallis were used to compare the pressure on each sensor at each turning angle. Based-line pressure data was recorded prior to any skiing. Consent to publish was acquired for the case study.

**Results**

A table will be presented which offers average pressures in the different sensors compared by inside/outside foot T-test and Wilcoxon agree on highly significant differences between inside and outside foot on sensors 1, 5 and 6. ANOVA found highly significant differences on outside turns on the sensor 1 (F = 12.74, p = 0.025 **) and 3 (F = 7.361, p = 0.0154 *). Being in both cases higher the pressure found on 60 degrees turn. Kruskal-Wallis shown highly significant differences only in sensor 1 (Kruskal-Wallis chi-squared = 8.433, p = 0.015 ***).

**Discussion and conclusion**

As single case study the results should be interpreted cautiously. However from this observational study it is evident that outside foot has higher pressures than inside foot on sensors 1, 5, 6. More closed turns (60°) produce higher pressures on outside foot on sensor 1 and 3. Data from leg sensors was inconclusive.

## P8 If the shoe fits: a footwear choice toolkit informed by social science methodologies

### Lisa Farndon^1^, Vicki Robinson^2^, Emily Nicholls^2^

#### ^1^Sheffield Teaching Hospitals NHS Foundation Trust, Sheffield, UK; ^2^Sociological Studies, University of Sheffield, Sheffield, UK

##### **Correspondence:** Lisa Farndon - Sheffield Teaching Hospitals NHS Foundation Trust, Sheffield, UK

**Background**

Previous research highlighted the centrality of footwear to individuals' sense of their identity, demonstrating that shoes must 'fit' someone socially, as well as functionally. However, unhealthy shoes can have a detrimental effect on foot health and mobility.

**Methods**

This project adapts qualitative social science methods to enable podiatrists to understand the broader contribution of footwear to patients' sense of themselves. Semi-structured interviews were conducted with 6 podiatrists/shoe-fitters and 12 people with foot problems.

**Results**

These identified four distinct themes which were barriers when selecting appropriate footwear; practicalities, personal, purpose and pressures. There can be conflict in what podiatrists and the people that they support and treat look for in a shoe, with many service users raising the importance of the visual appearance of footwear and links between footwear, occasion and identity. These themes were combined to form a toolkit. Shoe diaries, follow up interviews and a focus group with practitioners and service users further refined the toolkit.

**Discussion and conclusion**

This online toolkit is available to support foot health professionals to ‘make every contact count’ and help them to identify and address patients’ barriers to making healthier shoe choices in the hope that positive, long-term and sustainable footwear changes are made.

## P9 The identification of emotions from gait

### Tabitha Birch^1^, Ivan Birch^2^

#### ^1^University of Roehampton, London, UK; ^2^Sheffield Teaching Hospitals NHS Foundation Trust, Sheffield, UK

##### **Correspondence:** Ivan Birch - Sheffield Teaching Hospitals NHS Foundation Trust, Sheffield, UK

**Background**

Anecdotal evidence would suggest that it is possible to identify the occurrence of strong emotions from their effect on the gait.

**Aim**

As the use of forensic gait analysis continues to increase for both evidential and investigative purposes, the possible effects on gait of intrinsic factors such as emotion need to be understood by practitioners working in the field. This study investigated the ability of 30 untrained analysts to identified emotions from gait.

**Methods**

A previously validated and published methodology was used to video 3 male and 3 female walkers, while displaying one of four emotions (pride, happiness, fear and anger) or no emotion. Each walker was filmed displaying each of the emotions and no emotion, from the front, back, left and right sides, and obliquely. The analysts were individually shown the 30 sets of footage and asked to identify which, if any, emotion was being displayed. They were also asked to record their confidence in their decision using a validated scale widely used in forensic disciplines.

**Results**

The untrained analysts correctly identified all four emotions and no emotion on significantly more occasions than would have been predicted by chance (p < .01). The correct identification of anger was associated with ‘strong’ confidence (p < .01), while that of the other three emotions and no emotion were associated with ‘moderate’ confidence (p < .01).

**Discussion and conclusion**

The results suggest that the effects of emotions on gait can be detected by untrained observers from video footage, and should therefore be taken into consideration during the process of forensic gait analysis. The results also have implications for forensic psychology, the effects on gait possibly providing an aid to establishing the emotional state of a suspect prior to, during and after an offence has been committed, and therefore helping to inform sentencing.

## P11 Experience of foot problems in patients with systemic lupus erythematosus

### Simon Otter^1^, Sunil Kumar^2^, Peter Gow^2^, Nicola Dalbeth^3^, Michael Corkill^4^, Kevin Davies^5^, Sam Panthakalam^6^, Maheswaran Rohan^7^, Keith Rome^7^

#### ^1^University of Brighton, Brighton, UK; ^2^Counties Manuaku District Health Board, Auckland, NZ; ^3^Auckland District Health Board, Auckland, NZ; ^4^Waitemata District Health Board, Auckland, NZ; ^5^Brighton & Sussex Medical School, Brighton, UK; ^6^East Sussex Healthcare Trust, Eastbourne, UK; ^7^AUT University, Auckland, NZ

##### **Correspondence:** Simon Otter - University of Brighton, Brighton, UK

**Background**

Foot complaints are common in inflammatory arthropathies such as rheumatoid arthritis and cause considerable disability. However, little is published about the nature and extent of foot complaints in systemic lupus erythematosus (SLE).

**Aim**

The objective is to explore clinical features and symptoms of foot involvement in people with SLE from the patients’ perspective.

**Methods**

We developed and tested a new 40-item item self-administered questionnaire, using a five-stage development process utilising patient involvement throughout to ensure face and content validity. The self-administered instrument was posted to 406 people with SLE attending adult rheumatology clinics across three health boards in Auckland, New Zealand. The questionnaire enquired about symptoms of foot pain, extra-articular features, anatomical distribution of symptoms according to validated foot-mannequins and the impact of foot symptoms on activities of daily living and well-being.

**Results**

From 131 responses (32 % response rate), 89 % women, mean (SD) age 51 (15.1), mean (SD) disease duration 12.5 (11.1) years. 77.1 % respondents experienced foot pain during the course of their lupus, 51.9 % reporting pain in the last month and 45 % reporting current foot pain. The mean (SD) score on a 10 cm visual analogue scale for current foot pain was 4.9 (2.2). There was no association between foot pain and the following variables: age, duration of SLE, body mass index, ethnicity, work status or smoking. Females were significantly more likely to report foot pain (p = 0.028) and being female was a risk factor for foot pain independent of all other demographic variables (OR 1.78 [95 % CI 0.62-2.98]). All regions of the feet were affected, with the hindfoot and ankle most frequently affected during the course of the disease (32 % and 30 % respectively). Extra-articular symptoms (cold feet, Chilblains, Raynaud’s phenomenon and numbness were common. Respondents reported foot pain prevented sleep for 36 % and 33 % reported pain in their feet had a negative effect on their emotions. Life in general was adversely affected by foot pain with 60 % reporting limitation in foot-related activities of daily living (e.g. walking). Taken together, 70 % of respondents reported foot problems that interfered with social activities and 60 % reported foot complaints that interfered with their family activities.

**Conclusion**

Foot symptoms in SLE are common, heterogeneous in nature and may have a negative impact on patient well-being.

## P14 Negative pressure wound therapy for the management of foot wounds in the diabetic population: a review of the literature

### Chloe Egan^1,2^, Lisa Chandler^2^

#### ^1^Cambridgeshire and Peterborough NHS Foundation Trust, Cambridge, UK; ^2^University of Northampton, Northampton, UK

##### **Correspondence:** Chloe Egan - Cambridgeshire and Peterborough NHS Foundation Trust, Cambridge, UK

**Background**

Diabetes-related foot complications place a significant burden on the National Health Service (NHS) and the economy. The most common and costly sequelae of the disease includes ulceration, gangrene, infection, and ultimately amputation. Research suggests that these complications often have detrimental psychosocial effects on individuals who suffer with them. The management of diabetic foot wounds is a continuing battle for health services and therefore any intervention that promotes healing and possibly prevents the need for amputation could be extremely valuable.

One such intervention currently being used throughout the NHS and globally is Negative Pressure Wound Therapy (NPWT), a technique that uses negative pressure to remove fluid from open wounds through a specialised sealed dressing placed into the wound cavity. The mechanism by which NPWT works is not yet fully understood but it is thought to be associated with the removal of exudate, increased blood supply to the area, and reduction of cytokines and matrix metalloproteinases.

**Aim**

The objective of this review was to ascertain the effectiveness of NPWT as a treatment for diabetic foot wounds and consider the cost effectiveness of this intervention.

**Methods**

A systematic search of the literature was carried out using a set of relevant key words to identify available literature published between January 2005 and January 2015. Ten primary research studies were identified as suitable for inclusion following critical assessment using the Critical Appraisal Skills Program (CASP) appraisal tools, the CONSORT statement, and other appropriate frameworks.

**Relevance/impact**

Despite the increasing use of NPWT there is an apparent lack of relevant and robust Randomised Controlled Trials (RCTs) to justify this. There is a large volume of literature regarding the use of NPWT for various wound types, but very few studies specific to foot wounds in the diabetic population. Furthermore, most of the studies that do exist include case studies, small cohort studies, and poor quality RCTs. Due to the promising results seen in the widespread use of NPWT by health services it seems that the gap in relevant research regarding this technology should be considered further.

**Results**

The findings of this review indicate that NPWT has a positive effect on wound healing including reducing time to healing. However, the studies currently available are few and of varying quality, many with methodological flaws. Despite increased material costs compared to traditional wound care methods, research suggests that NPWT has the potential to lower care costs overall due to a reduced need for in-patient care.

**Discussion and conclusion**

The need for further quality RCTs to assess the effectiveness of NPWT for the management of diabetic foot wounds is highlighted. The onus should be on large numbers of participants, robust patient relevant outcome measures, and mid to long term studies with objective outcome measures to provide evidence of clinical effectiveness. It is proposed that future research should focus on developing a set of national guidelines for the use of NPWT in diabetic wound management.

## P15 Lower limb vascular assessment in diabetes: a multifaceted assessment of objective screening techniques

### Peta Tehan, Vivienne Chuter, Jennifer Sonter, Sean Lanting

#### University of Newcastle, Newcastle upon Tyne, UK

##### **Correspondence:** Peta Tehan - University of Newcastle, Newcastle upon Tyne, UK

**Background**

Diabetes-related lower limb macro- and microvascular disease is associated foot complications including ulceration and amputation1. The nature of this multi-system disease process makes accurate vascular examination difficult. This study evaluated the reliability and diagnostic accuracy of the ankle-brachial index (ABI), toe-brachial index (TBI) and continuous wave Doppler (CWD) assessment in people with diabetes and determined the strength of the relationship of these with history of foot complications.

**Methods**

Ankle and brachial pressure measurements and CWD assessment were performed using handheld Doppler and toe pressures using photoplethesmography. Retesting was performed 7–10 days later. Sensitivity and specificity of the ABI and TBI were determined using colour duplex ultrasound as reference standard and receiver operating characteristic (ROC) analysis was performed to assess the clinical utility of the ABI and TBI. Logistic regression was used to determine the relationship between vascular measurements and history of foot complications.

**Results**

A total of 389 participants with diabetes were recruited to the various arms of this study. Reliability of CWD was generally poor-to-moderate ranging from k = 0.17 (95 % CI −0.15 to 0.49) to k = 0.44 (95 % CI: 0.03 to 0.88). Reliability of the TBI and ABI were excellent ranging from ICC 0.75 (95 % CI −0.19 to 0.28) to 0.81 (95 % LOA −0.23 to 0.25). CWD had the highest sensitivity and specificity for detecting PAD (74 % and 93 % respectively). ROC analysis demonstrated the TBI had greater clinical efficacy for the diagnosis of PAD (ROC area: 0.75 p = 0.0001) than the ABI (ROC area: 0.58, p = 0.27). Participants with a TBI of less 0.6 were almost eleven times more likely to have a history of foot complication (OR: 10.73,p = 0.048). The ABI and CWD were not independently associated with history of foot complications.

**Conclusion**

CWD has the highest sensitivity and specificity for PAD however had poor clinical reliability. The TBI and ABI both demonstrated acceptable reliability however the TBI was more effective at detecting PAD, associated with elevated likelihood of foot complications and may be a more effective vascular test in people with diabetes.

**References**

1. Boulton AJM, Vileikyte L, Ragnarson-Tennvall G, Apelqvist J. The global burden of diabetic foot disease. Lancet. 2005;366(9498):1719–1724.

## P16 Improving outcomes for diabetes foot complications

### Lorna Hicks

#### Betsi Cadwaladr University Health Board, Bangor, Wales

**Background**

Diabetes has a reputation for causing complications that may increase a persons’ likelyhood of lower extremity amputations (LEAs).

There was a history of high numbers of amputations among the diabetes population in the area of North Wales served by the general hospital.

The diabetes consultant and specialist nurse reviewed notes of patients to identify the amputations and assess the patient pathway. Two issues were identified; the absence of a diabetes specialist podiatrist with extended scope skills and a multidisciplinary team (MDT) approach to management and prevention. In 2003 this was rectified.

Our study was a retrospective review of the LEAs among diabetes patients over a 5 year period compared with the 5 year data prior to MDT development.

**Methods**

Intervention: A podiatrist was employed, to work with the acute diabetes team and develop a MDT, with a target of reducing LEAs and admissions for diabetes patients who have foot complications. Based in renal and diabetes outpatients and integrated with the ward.

A specific, weekly, diabetes foot clinic was established. The consultant and podiatrist would jointly assess the needs of the patient. It accepted referrals for new/acute diabetes foot complications; mainly ulcers and Charcot foot.

Strategy for change: In the initial months/years several audits and reviews were completed to allow better targeting of resources. These include 1) we identified that 50 % of clinic attendees had vascular issues- we initiated MDT vascular clinics. 2) A specific geographical area produced most of the referrals- fed back to the community , a new podiatrist post was funded to target diabetes foot health as part of a ‘spend to save’ strategy. 3) Re ulceration associated with poor footwear fit – an orthotist joined the MDT monthly.

At their first visit patients had their general health cardiovascular risk factors and diabetes management reviewed; foot complications are often the first presenting complication however other complications may be hidden and would contribute to poor outcome.

Measurement of improvement: We investigated ulcer healing rates, length of hospital stay and amputation rates.

**Results and discussion**

We were able to demonstrate the intervention had achieved a reduction in lower limb amputations for the diabetes population. Average healing rate of 8 weeks. Reduced length of hospital stay. Amputations reduced by up to 50 % (range10-19) compared to pre MDT data (range13-28) this against a background of increasing numbers of diagnoses of diabetes.

**Conclusion**

Recommendation: treat the whole of the patient not just the hole in the foot to produce improved outcomes when managed through a multidisciplinary clinic.

## P17 Acupuncture… an alternative or adjunctive treatment option for diabetes-related neuropathic pain?

### Christopher Joyce^1^, David Watterson^2^, Caroline McIntosh^1^

#### ^1^National University of Ireland, Galway, Ireland; ^2^Health Service Executive West, Galway, Ireland

##### **Correspondence:** Christopher Joyce - National University of Ireland, Galway, Ireland

**Background**

Global prevalence of diabetes is increasing at an alarming rate with one in three Irish people having a family member with diabetes. With increased prevalence comes an increase in complications and neuropathic pain is an all too common problem amongst people with diabetes. It is exceptionally challenging to treat and despite a range of pharmaceutical options many patients live with this pain which can severely impact on their health related quality of life and wellbeing.

Acupuncture, a therapeutic modality involving the insertion of fine needles, in western medicine is thought to act mainly by stimulating the nervous system. It's most widespread application is for pain relief – musculoskeletal pain and other forms of chronic pain such as neuralgia and cancer pain. Although acupuncture has been used and cited as a potential therapy for pain relief, the current evidence base to support its use for neuropathic pain, in particular, is lacking.

**Methods**

A convenience sample of 12 participants (8 male and 4 female), with diagnosed Diabetes Mellitus (3 Type 1 and 9 Type 2) with a history of diabetes-related neuropathic pain were recruited. Demographic information, Leeds Assessment of neuropathic symptoms and signs questionnaire, Ankle-Brachial Index readings, neurothesiometer readings, Visual analogue scale (VAS) for presenting pain, VAS for belief in acupuncture therapy and selected Neuro-Qol questionnaire data was collected on the 1st visit. Participants were then given 5 acupuncture treatments over 4 weeks on both feet, points used were Bafeng and Taichong (5 points per foot). Acupuncture needles were inserted for 20 minutes, with no manipulation conducted. VAS for pain and belief and selected Neuro-Qol questionnaire data was collected after the 5th session. Paired-samples T-test was used to test for statistical significance.

**Results**

No significant difference was found (p > 0.082) in pain levels before and after a course of acupuncture. The male gender (n = 8) responded well to the therapy, and showed statistical significant (p < 0.04) but no significance in woman. Perceived belief in acupuncture among patients was statistically significant (p < 0.011) after 4 weeks of therapy. Neuro-Qol results showed improvements in all areas (Satisfaction, Ability, Depression, Fatigue , Sleep disturbance and Lower extremity mobility) however, depression, fatigue and sleep disturbances were the only to show significant improvements in participants (p < 0.014); no differences in gender where found.

**Conclusion**

This study highlights that the use of acupuncture can significantly reduce pain experienced by people with diabetes-related neuropathic pain and should be considered as an adjunctive therapy in conjunction with current evidence-based medicines and therapies for diabetes-related neuropathic pain. Consideration for this treatment modality should be used by podiatrists trained in this skill and other health professionals who have the ability to refer their patients for this treatment. Further work is required in the use of longer-term and possible placebo-controlled trails to investigate this studies results, however until true verum acupuncture is understood in western medicine, a true placebo controlled randomised trail is not possible.

## P18 “My back is in agony” – A cross-sectional study into the relationship between musculoskeletal complaints and a whole body postural risk assessment in podiatry students

### Christopher Joyce, Nigel Roberts

#### National University of Ireland, Galway, Galway, Ireland

##### **Correspondence:** Christopher Joyce - National University of Ireland, Galway, Galway, Ireland

**Background**

11.9 % of the Irish workforce has been clinically diagnosed with a musculoskeletal complaint and is thought to cost the Irish taxpayer €750 million a year. Correct assessment and analysis of job posture can identify potential areas of developing musculoskeletal complaints. Due to an increase in podiatric services across the world, assessing and preventing musculoskeletal complaints in podiatry students before they occur can reduce the monetary strain on the taxpayer and allow the upkeep of vital podiatric services to the general public.

**Methods**

A quantitative, purposive sampling, cross-sectional approach was utilised. All data collection took place within Merlin Park Podiatry Clinic, where all eligible students completed their clinical training. 65 participants were enrolled in the study. Demographics were collected, musculoskeletal complaints via the Nordic Musculoskeletal Questionnaire and job posture recording using the slow-motion analysis tool Ubersense application on the iPad, with posture scoring and classification based on the Rapid Entire Body Assessment (REBA) scoring tool.

**Results**

In this predominately female (81.5 %) cohort with a mean age of 22(±5.04), 98 % of participants noted at least 1 musculoskeletal complaint via the Nordic Musculoskeletal Questionnaire and a mean postural score by Rapid Entire Body Assessment of 6.96(±2.11), indicating their posture has the potential to induce future musculoskeletal complaints. A modest, positive relationship was found between musculoskeletal complaints and job posture (r = 0.556), however significance was not reached (p = 0.249). Unlike other studies in this field, a significant association was noted between males and the prevalence of musculoskeletal complaints (p = 0.039).

**Conclusion**

Musculoskeletal complaints are highly prevalent in Irish podiatry students and though no significance was found between these and job posture, the high incidence of poor posture in these students needs attention. Further investigation is required in this field, especially through the use of longitudinal studies or randomised controlled trails to find causality rather than relationship between musculoskeletal complaints and job posture.

## P19 Swabs of the treatment couches: Does the material type and texture of podiatric treatment couches increase microorganism contamination?

### Jacqueline Forss, Chrystalla Charalambous, Jack Kirby, Oluwakemi Ojo

#### University of Brighton, Brighton, UK

##### **Correspondence:** Jacqueline Forss - University of Brighton, Brighton, UK

**Background**

Cross-infections are a significant contributor to cost; morbidity and mortality associated with hospital treatment and may be brought about by a diversity of microorganisms. Once infection has occurred, the source of nosocomial pathogens often remains elusive, but is sometimes traced back to contaminated environmental surfaces and clinical equipment, including treatment couches. (Ohl et al., 2012, Woodland et al., 1998).

**Aim**

This study aims to improve our understanding of how the material type and texture of podiatric treatment couches may affect bacteria transmission.

**Methods**

Eight podiatric treatment couches were selected from an out-patient podiatry clinic – specifically 4 had a smooth grain finish and four a rough grain finish. Three swabs were taken from each chair, two 5 cm^2^ areas of the couch leg rest and seat area, plus a swab was taken of the leg rest adjustment handle. A template ensured the same portion of each couch was sampled. Each couch was sampled at the end of a clinical session following the recommended in-house post –cleaning protocol. Samples were cultured in agar plates by the microbiology department at 37 °C for 48 hours followed by colony counts and organism identification. To facilitate organism recognition, gram staining, DNase testing, oxidase testing and organism appearance was undertaken.

**Results**

Bacterial contamination was present on five of the eight leg rests and, three on the handles and one seat area. The bacterial present included environmental gram-negative rods, coagulase-negative Staphylococcus, Klebsiella, Micrococcus, Aerococcus species and Diptheroid species bacteria. Colony counts were small overall ranging between zero and eight. Seat areas had the lowest growth of bacteria (1 colony) followed by the leg rests (1–2 colonies). The smooth grain finish revealed greater bacterial growth than the rough finish. However, adjustment handles demonstrated the largest number and type of bacteria (up to 8 colonies).

**Conclusion**

The material type and texture of podiatric treatment couches did not suggest a demonstrable effect on bacterial contamination. However, the role of adjustment handles in the transmission of pathogens is worthy of further investigation. It is possible couches are cleaned and then re-contaminated by the practitioner when adjusting the leg rest. Including this aspect of practice as part of the safeguards for prevention of bacterial cross-contamination would be warranted.

## P20 Does increased exudate viscosity effect the absorption rate of exudate into four different wound dressings?

### Jacqueline Forss, Sarah Caukill, Jacqueline Capon, Radiance Fong, Louis Loy

#### University of Brighton, Brighton, UK

##### **Correspondence:** Jacqueline Forss - University of Brighton, Brighton, UK

**Background**

Exudate plays an important role in wound healing as it contains a variety of substances including water, electrolytes, nutrients, imflammatory mediators, whitecells, proteases,growth factors and waste products. The presence of infection increases the viscosity of wound exudate due to changes in bacterial load and protein content. There is limited work examining rates of exudate absorption into dressings and the studies which have been conducted have used saline solution to represent wound exudate, however saline is different in both viscosity and composition to they various types of exudate produced from chronic and infected wounds.

**Aim**

To examine the effect of three different artificial exudate viscosity on the rate of absorption into four classes of wound dressings.

**Methods**

The rate of absorption was measured on four different types of dressings following the protocol outlined by the British Standards test methods for primary wound dressings (BS EN 13726–1,2002).

Three test solutions of known viscosity were tested with: i - Saline solution to represent low viscosity exudate, ii- diluted mixture of 'Resource ThickenUp Clear' (Nestle Health Science) and saline to represent medium viscosity exudate and iii- More concentrated mixture of 'Resource ThickenUp Clear' and saline to represent high viscosity exudate.

Two way analysis of variance (ANOVA) was used to analyse the data.

**Results**

There was a significant statistical difference between solution viscosity and the rate of absorption across all dressing types, P = 0.001. The rate of absorption was shown to decrease as viscosity increased.

**Conclusion**

Viscosity increases the rate of absorption into the wound dressings tested. This could have a detrimental effect on the wound environment, by allowing higher viscosity exudates within infected wounds to remain on the wound surface for longer than previously anticipated.

## P21 An investigation into the microbial load of a 40 °C and 60 °C wash

### Matthew Diment, Madeleine Murray, Mairghread Ellis, Carla McArdle

#### Queen Margaret University, Musselburgh, Scotland

##### **Correspondence:** Matthew Diment - Queen Margaret University, Musselburgh, Scotland

**Background**

Over 4 million patients are estimated to acquire a healthcare associated infection (HCAIs) in the EU each year (The European Centre for Disease prevention and control, ECDC, 2015). Approximately 20-30 % of these infections can be prevented by intensive hygiene and control programmes (ECDC, 2015). Therefore maintaining a clean environment, wearing a clean uniform and adhering to current hand washing procedures are imperative to the prevention of HCAIs. Current guidelines regarding the washing of healthcare uniforms would suggest that 60 °C is the correct temperature to use however there remains to be a lack of evidence to support these guidelines and some inconsistencies with other guidelines (Scottish Government, 2010; The Health and Safety Executive, 2013).

**Aim**

The aim of this study was to identify the effect water temperature has on microbial load during a machine wash cycle of clinical uniforms comparing a 40 °C wash and a 60 °C wash.

**Methods**

Material was collected from clinical uniforms and 10×10cm squares were cut from the upper leg of the trousers. All materials used were sterilized prior to the experiment and aseptic techniques used at all times to minimise airborne contamination. In order to investigate the microbial load at 40 °C and 60 °C, materials were individually contaminated with a range of organisms pre wash, they were then washed at both temperatures and the microbial load post wash determined using selective agar plates (Trypticase Soy Agar and Sabouraud Agar). The organisms used included gram positive (Staphylococcus aureus) and gram negative bacteria (Escherichia coli), fungi (Absidia corymbifera) and yeast (Saccharomyces cerevisiae). All experiments were carried out in triplicate and two washes were performed for the testing of each individual organism. In addition a standardised washing detergent was used for all experiments.

**Results**

There was no difference in the level of contamination after materials were washed at 40 °C and 60 °C. Both washes displayed nil contamination at 40 and 60 °C as materials were described as ‘clean’ post wash.

**Discussion**

Washing at both temperatures displayed complete decontamination of all organisms post wash. In relation to HCAIs, this study demonstrates that these temperatures are enough to eradicate the bacterial contaminants that may present on the clinician’s uniforms at the end of their working day. This research data can potentially provide much needed evidence to facilitate the standardisation of current guidelines for the washing of clinical uniforms.

**Conclusion**

While larger studies investigating organisms cultured specifically from clinical environments need to be carried out, this study nevertheless demonstrates that washing at 40 °C and 60 °C are equally sufficient for the eradication of contamination on the surface of clinical uniform. This therefore questions the accuracy of guidelines which stipulate that clinical uniforms should be washed at 60 °C.

**Ethical approval statement**

Ethical approval was granted from Queen Margaret University divisional research ethics committee.

**Acknowledgements**

Recognition is given to the research team: Amanda Cork, Matthew Diment, Lena McEwan, Madeleine Murray, Kirsty Scott and Elenii Tofalidou.

## P23 The sensitivity and specificity of the toe brachial index in detecting peripheral arterial disease: a systematic review and meta-analysis

### Peta Tehan^1^, Vivienne Chuter^1^, Christopher Oldmeadow^2^

#### ^1^University of Newcastle, Newcastle upon Tyne, Tyne and Wear, UK; ^2^Hunter Medical Research Institute, 1 Kookaburra Circuit, New Lambton Heights NSW, Australia

##### **Correspondence:** Peta Tehan - University of Newcastle, Newcastle upon Tyne, Tyne and Wear, UK

**Background**

Peripheral arterial disease (PAD) has been estimated to affect up to 20 % of the adult population and can ultimately lead to the development of wounds, gangrene and amputation. With PAD sufferers being mostly asymptomatic, the condition is highly unrecognised; therefore routine clinical assessment of lower limb arterial flow in patients deemed at risk is essential in early detection and monitoring of the disease. The toe-brachial index (TBI) is used as an adjunct to the ankle-brachial index (ABI) for vascular screening. With increasing evidence suggesting the flaws in the ABI, particularly in specific populations, such as people with diabetes, the TBI is being used more widely.

**Methods**

A database search was conducted to identify current work relating to the sensitivity and specificity of toe brachial indices up to May 2013 using Ovid Medline (1946–2013), CINAHL Plus (1982 – 2013), Science Direct and PubMed. A bivariate model was used to analyse pairs of the logit (log odds) of sensitivity and logit of specificity. Summary measures such as the mean sensitivity and specificity, the diagnostic odds ratio, and Receiver operating characteristic (ROC) curve and the area under the curve were calculated. P-values and confidence intervals for the estimates were derived based on a normal distribution with a d.f of 1000.

**Results**

Six studies met the inclusion criteria. Sensitivity was reported in all six papers and ranged from 45 % to 100 %. Mean sensitivity was 0.77 (95 % CI 0.53 to 0.91, p = 0.028). Specificity was reported by five papers only and ranged from 76 % to 100 % specific. Mean specificity was 0.83 (95 % CI 0.75 to 0.89, p < 0.0001). Diagnostic odds ratio 16.7 (95 % CI 5.69 – 48.76, p = <0.0001) and AUC (0.86) indicated good test performance.

**Conclusions**

The TBI is a valuable adjunct to a non invasive vascular assessment and has the potential to identify PAD in specific populations, such as people with diabetes, and end stage renal failure, which may otherwise not be detected by large vessel screening methods. More research however, needs to be conducted to ascertain whether or not the TBI is a valid measure for detecting PAD in the general population at risk of PAD.

## P24 Medicines management activities and non-medical prescribing within podiatry and physiotherapy: an integrative review of the literature

### Nicola Carey^1^, Karen Stenner, Heather Gage^2^, Jane Brown^3^, Peter Williams^2^, Simon Otter^1^, Ann Moore^1^, Jude Edwards^2^, Freda Mold^2^, Molly Courtenay^4^

#### ^1^University of Brighton, Lewes Rd, Brighton, UK; ^2^University Of Surrey, Guildford, UK; ^3^Liverpool John Moore University, 70 Mount Pleasant, Liverpool, UK; ^4^Cardiff University, Cardiff, UK

##### **Correspondence:** Nicola Carey - University of Brighton, Lewes Rd, Brighton, UK

**Background**

At a time of financial constraint in the NHS, non-medical prescribing by physiotherapists and podiatrists provides organisations with the ability to improve productivity and quality of patient care. However, there is little evidence regarding the ways in which these podiatrists and physiotherapists use non-medical prescribing (NMP) in their daily practice nor its impact on patient outcomes. This integrative review was conducted in order to evaluate activities related to medicines management and prescribing undertaken by physiotherapists and podiatrists, and explore how the NMP role has been implemented in practice.

**Aim**

We aimed to systematically identify, and critically appraise the current evidence regarding physiotherapy and podiatrist medicines management activities (MMA) and NMP and impact on service provision and patient care.

**Methods**

Electronic databases, using terms developed to identify MMAs and NMP across a range of roles, were searched from May 1985 to July 2014 for physiotherapy, and January 1968 to July 2014 for podiatry, including published and grey literature. No limit was placed on document type, design or quality and articles were accepted from any clinical speciality and healthcare setting. Data on the roles, clinical context, and patient outcomes in which MMAs and NMP were used were extracted and analysed descriptively, and quality appraised by 2 reviewers using a validated mixed methods appraisal tool [1].

**Results**

Overall sixty five physiotherapy and 15 podiatry articles were identified. Of these 27(45.1 %) from 2 (13.3 %) were empirical studies from physiotherapy and podiatry respectively. Articles were of moderate quality, from seven countries.

Podiatry literature was limited, largely descriptive, and collectively provided a chronological account of NMP legislative developments in the UK and Australia. Two small UK audits suggested medicines adherence, and quality of care improved when podiatrists adopted the NMP role.

Evidence from physiotherapy indicated variation in MMAs, involvement with medicines administration, providing advice re dosage and safety, and recommending new medicines. Patient satisfaction and outcomes of care were equivalent to traditional models of service provision, e.g. extended scope practice of medicines administration via injection therapy. However, pharmacology knowledge was found to be inadequate and unmet training needs identified.

**Conclusions**

Findings of the review identified MMA practices in physiotherapy and highlighted a lack of evidence regarding podiatric practice. Although there is some evidence reporting positive outcomes of extended scope physiotherapists, there is a lack of evaluation related to non-medical prescribing by physiotherapists and podiatrists that points to a need for urgent and rigorous investigation.

## A7.2 Non-invasive vascular assessment in the foot with Diabetes: Diagnostic accuracy of ankle brachial index, toe brachial index and continuous wave Doppler

### Peta Tehan^1^, Alan Bray^2^, Vivienne Chuter^1^

#### ^1^University of Newcastle, Newcastle upon Tyne, Tyne and Wear, UK; ^2^Vascular Health Care, East Maitland, NSW, Australia

##### **Correspondence:** Peta Tehan - University of Newcastle, Newcastle upon Tyne, Tyne and Wear, UK

**Background**

Peripheral arterial disease (PAD) is estimated to affect 21 % of people over the age of 65. Routine non-invasive vascular assessment of the lower limb is recommended as a screening tool in those at risk of the disease including older people and people with diabetes. This is essential for early diagnosis and instigation of appropriate risk factor modification to avoid lower limb complications including ulceration and amputation. Accurate on going monitoring of lower limb vascular status is of particular importance for people with diabetes. Diabetes-related PAD is more frequent, more severe and associated with increased risk of PAD compared to PAD in the general population and patients are frequently asymptomatic. In this population non-invasive lower limb vascular assessment is also known to be challenging due to the presence of co-existent complications such medial arterial calcification.

Continuous wave Doppler (CWD), the ankle brachial index (ABI) and the toe-brachial index (TBI) are the most frequently used non-invasive screening methods for PAD. However there is limited evidence assessing their comparative diagnostic accuracy in people with diabetes and without diabetes.

**Aim**

The aim of this study was to determine the sensitivity and specificity of CWD, ABI and TBI in a population with, and without diabetes.

**Method**

A convenience sample of participants who met current American Heart Association guidelines for lower limb vascular screening was recruited from a community podiatry clinic and a private vascular clinic in Newcastle, New South Wales. CWD waveforms were obtained from the right side of participants along with ankle-brachial indices and toe-brachial indices. Diagnostic accuracy of CWD, ABI and TBI was determined using colour duplex ultrasound (CFDU) as a reference standard.

**Results**

119 participants were recruited, seventy-three with diabetes and forty-six without diabetes. In the group without diabetes CWD demonstrated similar high sensitivity (84 %, 95 % CI 60 to 96) to TBI (83 %, 95 % CI 58 to 96), whereas the sensitivity of the ABI was comparably low (47 %, 95 % CI 24 to 71). In participants with diabetes, CWD had the highest sensitivity (74 %, 95 % CI 55 to 88) followed by TBI (63 %, 95 % CI 45 to 79) and ABI (45 %, 95 % CI 27 to 63). Equally highly specific in the diabetes group was ABI (92 %, 95 % CI 80 to 98) and CWD (92 %, 95 % CI 80 to 98) followed by the TBI (82 %, 95 % CI 66 to 92).

**Conclusion**

CWD waveform is more likely to detect the presence of significant PAD compared to ABI and TBI as diagnosed by CFDU in people with and without diabetes. However as none of the three tests demonstrated perfect accuracy as independent tests they should not be singularly relied upon for clinical diagnosis of PAD.

## A7.5 The efficacy of dressings on post nail surgery phenolised wounds

### Pamela Hindmoor

#### New College Durham, Framwellgate Moor Campus, Framwellgate, Durham, UK

**Background**

Toe nail pathologies often require surgical intervention in podiatric practice. However, due to the corrosive nature of phenol, excessive exudate is produced and healing time is prolonged. Historically paraffin based tulle (bactigas) has been one of the primary dressings of choice following nail surgery. However, adhesion to the nail bed can cause pain and trauma on removal and these factors prompted King (2003) to suggest its use as a primary dressing should be discontinued. In a review of published literature Davies (2010) concluded from 12 comparative randomised control trials that when used in the treatment of exuding wounds foam dressings were superior to hydrocolloid dressings in terms of exudate management, as well as being associated with less dressing related trauma and pain. This suggests that a foam dressing may provide a suitable alternative to the traditional paraffin based tulle as a primary dressing following nail surgery.

**Methods**

Study participants were recruited from a group of patients presenting for nail avulsion surgery. Participants were randomly allocated to one of two study groups and received either A: Bactigras (n = 39) or B: Aquacel® Foam (n = 38) as the primary post-operative dressing. Data collection comprised the assessment of wound healing variables, with additional qualitative input from the study participants.

**Results**

Preliminary data analysis indicates Aquacel® Foam dressings provide better capacity to retain exudate, a lower incidence of peri-wound maceration and wound bed adherence. The patients allocated Aquacel® Foam dressings experienced less pain (VAS scale) on dressing removal (Mann–Whitney U tests revealed statistical significance p = 0.01). Data suggests patients' perceived significantly less post-operative pain (p = 0.04) and complications (post op bleeding p = 0.025, infection/phenol flare p = 0.03) in the wound healing process in the group allocated Aquacel® Foam dressings.

**Discussion and conclusion**

With the need for clinicians to consider patient centred outcomes consideration needs to be given to problems associated with pain, wound trauma caused by the removal of dressings that adhere to the wound bed and maceration of the peri-wound due to excessive exudate. Appropriate dressing selection plays a key role in promoting and maintaining a wound environment that is conducive to healing. A number of trials have shown Aquacel® ExtraTM to be successful in the treatment of burns and wounds. In 2012 Eekhof et al's Cochrane review concluded that post operative interventions such as: antibiotics; manuka honey; povidone-iodine with paraffin; hydrogel with paraffin or paraffin gauze showed no significant differences on infection rates, pain or healing time. This study compared the efficacy of bactigras and Aquacel® Foam dressings on postoperative nail surgery wounds, where phenol matrixectomy creates a localised chemical burn. Aquacel® Foam dressings appear to positively influence the patient's perceptions of post-operative pain and provide a better capacity to retain wound exudates and a lower incidence of prolonged post op bleeding and infection. However there was no significant difference in wound healing times following nail surgery.

## B7.1 Cross-sectional study investigating the role of proximal and distal factors in the development of patellofemoral joint pain

### Craig Gwynne, Sarah Curran

#### Cardiff Metropolitan University, Western Ave, Cardiff, UK

##### **Correspondence:** Craig Gwynne - Cardiff Metropolitan University, Western Ave, Cardiff, UK

**Background**

Patellofemoral joint (PFJt) pain characterised by peri- or retro-patellar knee pain during activities that load the knee extensors and PFJt is the most common knee condition seen among physically active individuals. Considerable debate remains as to the multifactorial etiology of PFJt pain with proximal factors including altered frontal plane limb alignment and distal factors including abnormal foot loading distribution suggested to influence knee mechanics and play a role in the pathogenesis of this condition.

**Aim**

To investigate FPPA and plantar pressure during unilateral limb loading tasks to increase understanding of proximal and distal factors in the etiology of PFJt pain.

**Methods**

Frontal plane knee alignment and plantar pressure distribution were quantified simultaneously in thirty recreationally active participants diagnosed with PFJt pain and thirty non-injured controls (aged 18–40) during single limb squats to 60° of knee flexion. Barefoot plantar pressure variables (peak force [PF], force time integral [FTI] and contact area [CA]) were collected for nine foot regions using a pressure platform (Emed, Novel GmbH, Munich, Germany). The frontal plane projection angle (FPPA) captured by digital video cameras was used to assess knee alignment.

Between-group differences were tested using three separate two-factor mixed ANOVA (2 [Group] x 9 [Plantar region]). Independent t-tests (2-tailed) were used to compare group differences in FPPA. Pearson product moment correlation coefficients were used to test the relationships between FPPA and plantar pressure variables with significant group x region interactions.

**Results**

Mean FPPA was significantly greater in the PFJt pain group (−12.27°) compared to the uninjured group (−6.43°) (95 % CI = −9.59 to −2.09; P = 0.003; ES = 0.38). Significant group-by-region interactions were found for PF (F = 2.604; P = 0.024) and CA (F = 5.331, P = 0.005) with the PFJt pain group exhibiting smaller PF at the midfoot and hallux as well as decreased CA at the medial and lateral heel, the midfoot and the hallux compared to the uninjured group. Significant correlations were observed between FPPA and PF at the hallux (r = 0.248; P = 0.28) and between FPPA and CA at the hallux (r = 0.326; P >0.01), with increased FPPA (more negative values) associated with decreased PF and CA at the hallux.

**Discussion**

Individuals with PFJt pain demonstrate excessive frontal plane knee alignment exhibited as dynamic knee valgus during unilateral limb loading. In addition, PFJt pain subjects also present with smaller plantar loads of the medial forefoot that may indicate decreased foot pronation during similar tasks. This abnormal movement pattern could induce torque alterations within the lower kinetic chain and increase PFJt loading.

**Conclusion**

A possible association between abnormal knee alignment and decreased foot pronation in patients with PFJt pain may be addressed through combined rehabilitation programmes consisting of hip strengthening and foot orthoses.

## B7.2 Podiatrist’s interpretation and use of evidence in MSK practice

### Andy Bridgen

#### University of Huddersfield, Queensgate, Huddersfield, UK

**Background**

The development of podiatric biomechanics could be seen as part of a wider quest for legitimacy by the profession, allowing podiatrists to improve their professional status by claiming a body of knowledge and skills and enabling podiatrists to access a different group of patients. However, podiatric biomechanics theories and effectiveness of functional orthoses have been called into question, as there may be little research evidence to support them. There are many podiatrists who would argue with this statement and point to evidence that functional orthoses are an effective treatment for musculoskeletal conditions. These conflicting views of evidence have not affected the growth of the MSK specialism within podiatry.

**Aim**

The study explores MSK/ biomechanics podiatrists’ beliefs about evidence-based practice in their area. It discusses their perceptions of the interpretation of research and other forms of evidence. It appraises which types of evidence affect the clinical practice of MSK/biomechanics podiatrists.

**Methods**

This was a qualitative study which explored podiatrists’ beliefs about evidence-based practice in MSK practice, their perceptions of the interpretation of research and other forms of evidence. 17 in-depth interviews were conducted with podiatrists, 9 NHS, 6 private, 2 academics, who treat MSK conditions with functional orthoses. The data was analysed through a hermeneutic approach that using interpretative phenomenological analysis. This is less focused on phenomenological description, more on interpreting the data in a wider social, cultural and theoretical context.

**Results**

The participants understand the concept of evidence-based practice, as the application of quantitative research evidence to their practice. Research evidence cannot always be easily applied in practice due to the variances in orthotic design, in the causes of MSK conditions and the differences in patients’ lifestyles and preferences. Research evidence does inform their practice but they tend to interpret research evidence according to their own experiences. The participants value their clinical experience, which is formed by testing of orthotic materials and designs and patient responses to them. The evidence that they use could be described as a process of trial and error informed by their patient feedback. A lack of this experience may lead to inexperienced podiatrists’ being unsure about which treatments to give. Patient feedback was seen as the most important form of evidence. However, outcome data was collected only by some of the participants and this was rarely collated and analysed.

**Conclusions**

The participants may not be undertaking evidence based practice as they define it, as using research evidence in practice. They use their clinical experience more than research evidence in practice. They interpret and use research evidence through their own practical experience. Their experience is formed by a process of trial and error, with orthotic designs, and patient feedback about their treatments. The dependency of podiatric MSK practice on patient evidence rather than research has led to fears that podiatrists’ claims of legitimacy in MSK practice may be questioned. These fears may be allayed by more comprehensive evaluation of the efficacy of functional orthoses through the collation and analysis of outcome data. There also needs to be a wider debate in podiatry about the definition of evidence in evidence based practice and the types of evidence that are really utilised in practice.

## B7.4 Predictors of falling in older podiatry patients – findings from the REFORM study

### Caroline Fairhurst^1^, Joy Adamson^1^, Belen Corbacho Martin^1^, Sarah Cockayne^1^, Catherine Hewitt^1^, Kate Hicks^1^, Anne-Maree Keenan^2,3^, Lorraine Loughrey-Green^1,2^, Hylton Menz^4^, Anthony Redmond^2,3^, Sara Rodgers^1^, Jude Watson^1^, David Torgerson^1^, Robin Hull^5^, Sarah Lamb^6^, Caroline McIntosh^7^, Wesley Vernon^8^, Lisa Farndon^9^

#### ^1^York Trials Unit, University of York, Heslington, York, UK; ^2^NIHR Leeds Musculoskeletal Biomedical Research Unit, Chapel Allerton Hospital, Leeds, UK; ^3^Leeds Institute of Rheumatology and Musculoskeletal Medicine, University of Leeds, Leeds, UK; ^4^Faculty of Health Sciences, Lower Extremity and Gait Studies Program, La Trobe University, Bundoora, Victoria, Australia; ^5^Podiatry Services, Harrogate and District NHS Foundation Trust, Harrogate District Hospital, Lancaster Park Rd, Harrogate, UK; ^6^Nuffield Department of Orthopaedics, Rheumatology and Musculoskeletal Sciences, Kadoorie Critical Care Research Centre, John Radcliffe Hospital, University of Oxford, Headley Way, Oxford, UK; ^7^National University of Ireland, University Rd, Galway, Ireland; ^8^Podiatry Services, Sheffield Teaching Hospitals NHS Foundation Trust, Jordanthorpe Health Centre, 1 Dyche Close, Sheffield, UK; ^9^Podiatry Services, Sheffield Primary Care Trust, Fulwood House, Old Fulwood Road, Sheffield, UK

##### **Correspondence:** Caroline Fairhurst - York Trials Unit, University of York, Heslington, York, UK

**Background and aim**

Falls are a common and serious cause of morbidity in older people. Identifying risk factors for falling is necessary to design targeted interventions to lessen the risk of falls and falls related injuries. In the REFORM study we recruited a cohort of older people from routine podiatry clinics in Yorkshire, North Lincolnshire, Kent, South Tyneside, Stockton-on-Tees, Hartlepool, Southampton and Galway and followed them up for falls. We have investigated predictors of falling in this cohort.

**Methods**

2,309 eligible, consenting participants aged 65 years or over were recruited into the REFORM cohort. Data, including recent falls, fear of falling and medication use, were collected from the participants. The cohort is being followed up for prospective falls data via self-report falls calendars. Follow-up will conclude in December 2015. Logistic regression was used to investigate which variables collected at baseline are statistically significant predictors of having at least one fall over 12 months. We considered the percentage of patients considered at high risk of falls and the percentage that reported having been referred to a falls clinic in the past on entry to the cohort.

**Results**

To date, 1,566 (67.8 %) participants have been followed up for at least 12 months, of which 618 (39.5 %) have reported at least one fall. Having had a fall in the previous 12 months (OR 2.75, 95 % CI 2.16 to 3.48, p < 0.001) and worrying about falling at least some of the time (OR 2.03, 1.60 to 2.58, p < 0.001) were strong predictors of a fall in the next 12 months in this cohort (OR > 2).

On entry to the cohort, 101 (4.4 %) participants reported having been referred to a falls clinic or falls service in the past. Participants that had been referred were, on average, older than participants that had not been referred (mean (SD) age 79.2 (6.9) vs 76.6 (7.0)) and five times more likely to have fallen in the last 12 months (OR 5.3, 95 % CI 3.4 to 8.2, p < 0.001). They were also seven times more likely to report a fear of falling (OR 7.4, 95 % CI 3.6 to 15.3, p < 0.001). From the cohort, we estimate that approximately 68 % of over 65 s are at a high risk of falling, but only 6 % of these are referred to a falls clinic/service.

**Conclusion**

Relevance: Podiatrists could play an important role in identifying patients with an increased risk of falling by asking a few simple questions, and targeting them for falls prevention interventions, such as referral to a falls clinic or an assessment of the home environment for falls hazards; thus, reducing the number of falls and the burden of falls related injuries on the NHS.

Identifying participants in routine care, at podiatry clinics or elsewhere, who have an increased risk of falling allows these patients to be offered interventions designed to reduce their risk of falling and obtaining injuries which require medical attention at a cost to the NHS.

## B7.5 The REFORM study: Insole preference, requirements and compliance of podiatry patient’s aged 65 and over and at risk of falling

### Lorraine Loughrey-Green^1,2^, Sarah Cockayne^2^, Anthony Redmond^1^, Anne-Maree Keenan^1^, Sara Rodgers^2^, Lisa Farndon^3^, Wesley Vernon^3^, David Torgerson^2^, Caroline Fairhurst^2^, Jude Watson^2^, Hylton Menz^4^, Sarah Lamb^5,6^, Robin Hull^7^

#### ^1^Leeds Institute of Rheumatic and Musculoskeletal Medicine and NIHR Leeds Musculoskeletal Biomedical Research Unit, Leeds, UK; ^2^York Trials Unit, Department of Health Sciences, University of York, York, UK; ^3^Podiatry Services, Sheffield PCT, Jordanthorpe Health Centre, Sheffield, UK; ^4^La Trobe University, Melbourne, Australia; ^5^Nuffield Department of Orthopaedics Rheumatology and Musculoskeletal Sciences, University of Oxford, Oxford, UK; ^6^Warwick Clinical Trials Unit, University of Warwick, Coventry, UK; ^7^Podiatry Services, Harrogate and District NHS Foundation Trust, Harrogate District Hospital, Harrogate, UK

##### **Correspondence:** Lorraine Loughrey-Green - Leeds Institute of Rheumatic and Musculoskeletal Medicine and NIHR Leeds Musculoskeletal Biomedical Research Unit, Leeds, UK

**Aim**

To record insole data through the development and delivery of a UK specific randomised controlled trial providing a podiatry package of care for the prevention of falls.

**Background**

Falls are a common and serious cause of morbidity with high societal costs. The REFORM study indicates that 40 % of UK podiatry patients aged 65 and over will have fallen within 12 months.

A previous study showed a statistically significant reduction in the rate of falls over 12 months for patients given a multi-faceted podiatry intervention, which included insoles.

The REFORM trial has provided information on the insole component of this replicated and UK specific trial, highlighting the current and potential needs of this patient group.

**Methods**

Pilot phase: Improve compliance rates, reflect UK provisions and inform insole use for the main trial by piloting and recording patient and podiatrist’s’ insole preference.

Main trial: Record current insole use in the podiatry caseload of patients at risk of falling.

Record modifications made to the study insole based on clinical observations.

Measure insole compliance via questionnaire at three months.

Record insole related adverse events.

**Results**

Pilot phase: Of 31 patients piloted, insole preference was as follows: Formthotics (n = 5), Pressure Perfect X-Line insole (n = 9) and standard X-Line insole (n = 17). The results in addition to podiatrist consensus informed the use of a standard X-Line insole for the main trial with an optional reinforced version where longevity was of concern.

Main trial: Preliminary data indicated 63 (28.5 %) of 221 patients already wearing insoles. This ranged from simple insoles (n = 16, 25.4 %) prefabricated insoles with simple modifications (n = 13,20.6 %)/complex modifications (n = 3,4.8 %)/no modifications (n = 11, 17.5 %), or bespoke total contact insoles (n = 14, 22.2 %). No description was available for 6 participants (9.5 %).

Of 120 study insoles issued, 48 (57.6 %) required modification.

Of 493 patients recruited to the REFORM intervention, at 3 months, 74 % of participants were wearing the insoles at least a little of the time, and 58 % most/all of the time.

Only 16 adverse events were reported relating to insole use.

**Discussion and conclusion**

The data indicates that a small proportion of existing patients at risk of falling within the podiatry caseload are currently wearing insoles as part of their routine care. Over half of these are classed as ‘simple’ insoles with either minor or no podiatry modifications. On inclusion to the study, over half of study patients required modified insoles to improve foot function and address mechanical pathology. This suggests a possible unmet need for biomechanical assessment and insole therapy for patients at risk of falling within the current podiatry caseload.

Patient and podiatrists’ preference is for a cost effective and easily modified insole more commonly used within the UK.

Patient compliance is good at three months with minimal related adverse events.

## B7.6 A podiatry intervention to reduce falls in care home residents is feasible and demonstrates benefits: results from PIRFECT, a feasibility randomised controlled trial

### Gavin Wylie^1,2^, Zoe Young^2^, Brian Williams^3^, Frank Sullivan^4^, Hylton Menz^5^, Simon Ogston^6^, Jacqui Morris^3^

#### ^1^School of Nursing and Midwifery, University of Dundee, Dundee, Scotland; ^2^Department of Podiatry, Allied Health Professions Directorate, NHS Tayside, Dundee, Scotland; ^3^Nursing Midwifery and Allied Health Professions Research Unit, Stirling University, Stirling, Scotland; ^4^Department of Family & Community Medicine, University of Toronto, North York General Hospital, Toronto, Canada; ^5^Lower Extremity and Gait Studies Program, Melbourne VIC, Australia; ^6^Centre for Biomedical Science and Public Health, University of Dundee, Dundee, Scotland

##### **Correspondence:** Gavin Wylie - School of Nursing and Midwifery, University of Dundee, Dundee, Scotland

**Background**

Previous research has demonstrated effectiveness of a podiatry intervention in reducing falls among community dwelling older people [1]. The purpose of this project was to (i) establish the feasibility of implementing and testing this existing podiatry intervention in the CH setting, (ii) remodel the intervention and subsequent trial in light of the feasibility findings to suit the CH context, and (iii) estimate the effect size of the remodelled intervention on falls in a pilot randomised controlled trial (RCT). Here, we report the findings from the pilot RCT.

**Methods**

The pre-RCT feasibility work has been reported elsewhere [2]. The remodelled multifaceted podiatry intervention (MPI) consists of: foot orthoses provision (Formthotics™, Christchurch, New Zealand), footwear assessment and provision, ankle dorsiflexion/plantarflexion exercises, and toe flexor exercises. We subsequently conducted a two armed, rater blinded, pilot randomised controlled trial of the MPI with outcome assessment at 3, 6 and 9 months.

**Results**

Forty-three CH residents were randomly allocated to receive either the MPI or usual care. Median time to first fall (days) was longer in the MPI group compared to the control group (91 + 5.9 vs. 64 + 27.1), however this did not reach significance (Log Rank test, p = .41). A significant difference in the number of falls was found between the 2 groups, favouring the MPI, directly after the 3 month intervention (p = .05, 95 % CI −1.59 to .36; mean falls MPI group 0.62 + 1.1 vs. mean falls control group 1.24 + 1.8) which translated to a medium effect size on the number of falls (Cohen’s d = .43). This significance disappeared following multiple imputation for missing data (p = .08, 95 % CI −1.39 to .31). There was no significant difference between the groups in the number of falls at the end of the 6-month follow up (p = .89, 95 % CI −1.26 to 1.22; mean falls MPI group 0.77 + 1.6 vs. mean falls control group 0.83 + 1.6). This translated to a small effect size (Cohen’s d = 0.06). Documented adherence to the exercise component of the MPI was poor, with 35 % of participants attempting the exercises 3 times per week.

**Conclusion**

Despite poor documented adherence, this work demonstrates a beneficial effect of the MPI on the time to first fall, and a beneficial effect size at the end of the intervention period. Our results suggest that participants adhering and completing the intervention protocol will benefit most from the intervention. If documented adherence can be improved the MPI has the potential to reduce falls in the CH setting. Based on these findings, a definitive multicentre RCT using the intervention and methods developed in this study is feasible and warranted.

**References**

1. Spink MJ et al. Effectiveness of a multifaceted podiatry intervention to prevent falls in community dwelling older people with disabling foot pain: randomised controlled trial. BMJ. 2011 January 16;342:d3411.

2. Wylie G et al. Finding your feet : The development of a podiatry intervention to reduce falls in care home residents. J Foot Ankle Res. 2015;8:A7.

## C7.1 A survey exploring footwear habits in people with stroke and people with Parkinson’s

### Cathy Bowen^1^, Dorit Kunkel^1^, Mark Cole^1^, Margaret Donovan-Hall^1^, Ruth Pickering^2^, Malcolm Burnett^1^, Dan Bader^1^, Judy Robison^1^, Louis Mamode^1^, Ann Ashburn^1^

#### ^1^Faculty of Health Sciences, University of Southampton, Southampton, UK; ^2^Faculty of Medicine, University of Southampton, Southampton, UK

##### **Correspondence:** Cathy Bowen - Faculty of Health Sciences, University of Southampton, Southampton, UK

**Background**

Falls are common following neurological disease and ill-fitting shoes have been identified as a risk factor for falls. To date, the only relevant studies have focused on people with arthritis, diabetes or the general older population. This investigation forms the first phase of a larger study in gathering information exploring foot problems and choice of indoor and outdoor footwear for people with Parkinson’s (PwP) and people with stroke (PwS).

**Methods**

Five-hundred anonymous postal questionnaires related to footwear use were distributed to health professionals, leads of Parkinson’s UK groups and stroke clubs within the wider Southampton area (May 2014-May 2015). These collaborators were asked to hand out survey packs to people with a confirmed diagnosis of either stroke or Parkinson’s.

**Results**

324 surveys were completed (128 PwS; 196 PwP); 143 women: 178 men (2 missing); average age 71.9 (SD 10.2). Time since stroke PwS: 1 week - 32 years. Time since diagnosis PwP: 1 week – 25 years. Reported falls PwP: 0 – 7 per year (py) (mode:2py); PwS: 0-2py (mode:0py). Reported foot problems (PwS: 42 %; PwP:53.6 %). Reported footcare help (PwS:30 %; PwP:46 %).

The type of shoes most often reported worn by PwS indoors: flat lace-up shoe (29.7 %); athletic shoe/runner (12.5 %); Oxford shoe (10.2 %); Surgical/bespoke shoes (6.3 %). Slippers were low usage (1.6 %). The type of shoes most often reported worn by PwP indoors: slipper (47.4 %); moccasin (14.8 %); socks or barefoot (8.1 %). Flat lace-up shoes were low usage (4.6 %).

Problems with current shoes were reported by PwS : 31.3 %; PwP : 32.2 %. For PwS, decisions for purchasing indoor shoes are mainly based on grip (74.2 %), support (59.4 %), ease of fastening (57.8 %), fashion (48.4 %). For PwP, decisions for purchasing indoor shoes are mainly based on comfort (86.7 %), fit (79.6 %%), grip (52 %), ease of fastening (50 %).

**Conclusion**

Half of PwP and PwS report some form of foot problem. Only one third seek foot care help. PwP report twice as many falls per year as PwS and are more likely to wear slippers most often whilst indoors. This implies that better targeted advice on indoor footwear use for PwP is essential for falls prevention strategies.

## C7.2 Painful foot osteoarthritis; a common symptom in a common pathology?

### Peter McQueen^1,2^, Maxine Daniels^3^, Michael Doherty^4^, Nigel Arden^2,5^, Cathy Bowen^1,2,5^

#### ^1^University of Southampton, Southampton, UK; ^2^University of Oxford, Oxford, UK; ^3^Barts Health NHS Trust, London, UK; ^4^University of Nottingham, Nottingham, UK; ^5^Medical Research Centre, Southampton, UK

##### **Correspondence:** Peter McQueen - University of Southampton, Southampton, UK

**Background**

Epidemiological investigations of osteoarthritis have rarely considered the feet such that, understanding of impact and treatment of foot osteoarthritis is limited. What also remains unclear is whether diagnosis should be based on a pain experience or on the radiographic appearance of changes consistent with osteoarthritis in the feet.

The purpose of this study was therefore to investigative the prevalence of foot osteoarthritis relative to the prevalence of co-existing foot pain.

**Methods**

A random sample of 68 (age range: 69–93) returning female participants (baseline: n = 1003) from an established study (Chingford 1000 Women study http://www.chingfordstudy.org.uk) underwent foot x-rays.

Dorsoplantar and lateral view foot x-rays were taken and scored for presence of osteoarthritis by a Podiatrist using the Australian Foot Atlas criteria. The atlas scores five joints: first metatarsophalangeal joint, first cuneometatarsal joint, second cuneometatarsal joint, Navicular first cuneiform joint and talonavicular joint.

Osteophytes are scored as absent (0), small (1), moderate (2), severe (3) and joint space narrowing is scored as none (0), definite (1), severe (2), joint fusion at least one point (3). The case definition of osteoarthritis: radiographic osteoarthritis can be considered to be present if a score of 2 or above is documented fro either osteophytes or joint space narrowing from either the dorsoplantar or lateral view.

The case definition of foot pain was considered as a positive response to the question whether they had "experienced any foot pain within the past month”.

**Results**

Changes consistent with osteoarthritis were detected in one or more joints of either foot in 43 participants (63 %). Of those who were identified as having osteoarthritis, 12 % (n = 5) of participants described having one or more days of foot pain in the last month with 58 % (n = 25) describing no pain (30 % incomplete responses, n = 13). An additional 4 participants (n = 9) of those who didn’t experience pain in the last month said they had experienced more than one day of foot pain during their life.

**Conclusion**

The results of this study suggest a high prevalence of foot osteoarthritis among women in later life. This is higher than reporting in previous studies, however this may be due to the cohort being older.

Surprisingly a low prevalence of foot pain co-existed with radiographic foot osteoarthritis. The relevance of pain as a symptom in foot osteoarthritis may therefore be limited as it is not a common clinical presentation.

We would like to thank all the participants of the Chingford Women Study and Professor Tim Spector, Dr Deborah Hart and Dr Alan Hakim and Maxine Daniels for their time and dedication.

## C7.4 Clinical diagnosis of symptomatic forefoot neuroma in the general population: Delphi based recommendations

### Charlotte Dando^1,2^, Lindsey Cherry^1,2^, Cathy Bowen^1^

#### ^1^University of Southampton, Southampton, UK; ^2^Solent NHS Trust, Southampton, UK

##### **Correspondence:** Charlotte Dando - University of Southampton, Southampton, UK

**Aim**

To develop diagnostic criteria that has agreed expert consensus for the clinical diagnosis of forefoot neuroma.

**Objectives**

1) Complete a literature review to identify the range of clinical practice methods used to diagnose forefoot neuroma. 2) Complete a Delphi exercise to identify the range of clinical practice methods used to diagnose forefoot neuroma.3) Complete a Delphi exercise to determine the consensus of recommendations regarding the best ways to clinically diagnose forefoot neuroma. 4) Determine expert panel recommendations to inform development of a clinical pathway.

**Methods**

A Delphi approach was utilised to explore individual’s beliefs, knowledge and behaviours on clinician diagnosed forefoot neuroma to specifically answer the question ”What is the best way to clinically diagnose forefoot neuroma in the general population?” In total 17 health professionals including Podiatrists, Radiologist, Rheumatologist, Orthopaedic surgeons and Physiotherapists consented to participate in the study. The Delphi method consisted of 3 rounds of voting, either by rejecting or accepting criteria formulated by the panel. One final round encouraged the panel members to rank the strength of the criteria recommendations. The whole process was conducted through email.

**Results and discussion**

The results from the Delphi study revealed a set of themes related to clinical diagnosis of forefoot neuroma as location of pain, non weight bearing sensation, weight bearing sensation, observations, clinical tests and imaging. The themes have been integrated to formulate a clinical guide to diagnosis of forefoot neuroma.

**Conclusion**

The next development stage is to test the clinical diagnostic criteria within a clinical setting, that being a NHS podiatry service.

## C7.5 The development and implementation of a Clinical Quality Improvement Framework suitable for use in community services

### Nichola Stefanou

#### Sunderland University, Sunderland, UK

**Background**

Quality improvement within the NHS has attracted increased attention; specifically within community health services the ability to demonstrate levels of quality and improvement is under scrutiny. The Department of Health continues to reaffirm its commitment to quality via a number of seminal texts, as well as healthcare reform (DH 2012; DH 2013). The most widely-accepted definition of quality within healthcare is based on the principles of patient safety, effectiveness and patient experience (DH 2013). However, explanation remains ambiguous and the responsibility of local interpretation is left at the discretion of individual organisations.

The Kings Fund (2014) suggests little emphasis has been placed on the measurement of quality in community services, despite the recommendations for a national drive towards improvement made by the Francis Report (DH 2012) and the Hard Truths Report (DH 2014).

**Aim**

No system was in place to facilitate continuous quality improvement in relation to clinical standards, patient experience and efficiency within community health services. This doctoral thesis outlines a programme of work undertaken with the purpose of developing and implementing a Quality Improvement Framework suitable for use in community services. A key objective being the implementation of the framework across all professional groups which includes therapies (Podiatry), nursing, medicine, children’s services and support workers.

**Methods**

A participatory action research approach (Kemmiss & McTaggart 2000) was applied to undertake this programme of doctoral research. Five cycles of action research were conducted with specific themes of work emerging from each. Within the cycles, data was collected via workshops, informal discussion, interviews and personal reflection. Each cycle of action research; converging towards a greater understanding and improved solution to the problem in practice.

**Results and discussion**

The findings revealed the need for a systematic and continuous cyclical approach to improve quality within community services. The requirement to place patients central to the approach was highlighted in order to gain support from frontline clinicians. A four-stage framework (engage, measure, report and improve) was developed alongside a raft of improvement tools and an e-learning package. This framework was implemented successfully across 180 teams (including all podiatric services) and continues to be used within all community services to date.

An unanticipated benefit highlighted that the framework improved staff experience and general satisfaction, through the opportunity for engagement at a team and organisational level.

**Conclusion**

This research developed and implemented a quality improvement framework suitable for use within community services across all professional groups. The framework has yielded an increase in the standard of care delivered to patients; more than 80 % of the 180 participating teams have achieved quality scores of 90 % or over.

It provides a pragmatic solution for measuring and improving quality, and impacts positively on staff experience.

## C7.6 The REFORM study - methodological considerations in running a cohort randomised controlled trial within a podiatry patient caseload

### Sarah Cockayne^1^, Joy Adamson^1^, Caroline Fairhurst^1^, Catherine Hewitt^1^, Anne-Maree Keenan^2,3^, Sally Lamb^4^, Lorraine Loughrey-Green^1,2^, Caroline McIntosh^5^, Hylton Menz^6^, Anthony Redmond^2^, Sara Rodgers^1^, Wesley Vernon^7^, Jude Watson^1^, Lisa Farndon^7^, Belen Corbacho^1^, Robin Hull^8^, David Torgerson^1^

#### ^1^Department of Health Sciences, Faculty of Sciences, University of York, York, UK; ^2^NIHR Leeds Musculoskeletal Biomedical Research Unit, Leeds UK; ^3^Leeds Institute of Rheumatic and Musculoskeletal Medicine, Leeds, UK; ^4^Nuffield Department of Orthopaedics, Rheumatology and Musculoskeletal Sciences, Kadoorie Critical Care Research Centre, John Radcliffe Hospital, University of Oxford, Oxford, UK; ^5^School of Health Sciences, Áras Moyola, NUI Galway, Ireland; ^6^School of Allied Health, College of Science, Health and Engineering, La Trobe University, Victoria Australia; ^7^Podiatry Services, Sheffield Teaching Hospitals NHS Foundation Trust, Jordanthorpe Health Centre, Sheffield, UK; ^8^Podiatry Services, Harrogate and District NHS Foundation Trust, Monkgate Health Centre, York, UK

##### **Correspondence:** Sarah Cockayne - Department of Health Sciences, Faculty of Sciences, University of York, York, UK

**Aim**

The REFORM study uses a cohort randomised controlled trial design to evaluate the clinical and cost effectiveness of a podiatry intervention for the prevention of falls in older people. The aim of this abstract is to report the methodological considerations in running this type of design with a podiatry patient caseload.

**Background**

Falls and fall related injuries are a serious cause of morbidity and cost to society. As foot problems and inappropriate footwear may increase the risk of falls, podiatric interventions could play a role in reducing falls. The cohort randomised controlled trial is a novel design not previously used in a podiatry setting.

**Methods**

We chose to conduct a modified cohort randomised controlled trial to test whether this design would (1) assist recruitment, (2) minimise attrition rates in randomised participants and (3) allow us to recruit to further trials. Community dwelling podiatry patients over 65 years of age, who had attended a routine podiatry appointment, were invited to take part in the REFORM study. Patients were informed about the possibility of being offered a package of podiatry care aimed at reducing falls, however only those allocated to the intervention group were notified about their group allocation. Patients who returned a screening and baseline questionnaire and consent form were entered into the REFORM cohort to be followed up for prospective falls data via falls calendars. Only those who completed at least one falls calendar and reported one fall in the past 12 months or one injurious fall in the past 24 months, or fear of falling were entered into the REFORM trial.

**Results**

37,389 participants were mailed an invitation to take part in the study; 4,189 (11.2 %) returned a screening form, of which 968 (23.1 %) declined to take part and 666 (15.9 %) were ineligible. The remaining 2,555 (61.0 %) were sent a baseline questionnaire and blank monthly falls calendars; 2,309 returned a baseline questionnaire and at least one completed falls calendar, of which 689 (29.8 %) were immediately eligible for the REFORM trial and were randomised. A further 294 were randomised after becoming eligible as a result of having a subsequent fall. A total of 1,010 participants were randomised to the REFORM trial: 493 (48.8 %) to the intervention group and 517 (51.2 %) to the control group; 1,299 remained in the cohort. A total of 313 (12.3 %) patients withdrew from the cohort. The trial attrition rate is currently 7.3 % which is within the anticipated 10 % rate.

We have submitted an NIHR grant application to use this cohort for a trial evaluating an occupational therapist intervention for the prevention of falls. The NIHR have invited us to submit a grant application to build on existing HTA funded studies.

**Discussion**

This design was feasible, aided recruitment and attrition rates are as expected. No ethical issues were raised about the design. However, there was a high cost of following up ineligible members of the cohort. We are waiting to hear the outcome of our application for funding for the new trial.

## A31 Jewel in the crown: Exploring the factors contributing to the development and impact of foot problems in Systemic Sclerosis (SSc)

### Begonya Alcacer-Pitarch, Anthony Redmond, Maya Buch, Anne-Maree Keenan

#### ^1^Leeds Institute of Rheumatic and Musculoskeletal Medicine, Leeds, UK; ^2^Leeds NIHR Biomedical Research Unit, Leeds, UK

##### **Correspondence:** Begonya Alcacer-Pitarch - Leeds Institute of Rheumatic and Musculoskeletal Medicine

**Background**

Systemic sclerosis (SSc) is a connective tissue disease characterised by vasculopathy, immune activation and fibrosis. The multisystem nature of the disease has a wide-ranging impact on the patient’s physical and psychosocial health. While foot problems in patients with SSc have been previously described and the association with disability noted, the impact of such problems on the individual has yet to be quantified. The aim of this study was to identify factors, including foot pathology, that impact on quality of life (QoL) in SSc.

**Methods**

Structural equation modelling (SEM), a sophisticated statistical modeling technique was used to explore the multifactorial pathways, including foot involvement, impacting on QoL. The potential candidate factors that were included in the SEM were identified through two methods: i) a literature review with expert consultation; and ii) a case–control cross-sectional study of patients with SSc. Physical and psychosocial factors, including foot-specific factors were used for the SEM to predict the impact on QoL as measured using the disease-specific SSc QoL questionnaire.

**Results**

One hundred and twenty one patients with SSc were recruited (106 female; median age 59, ranges 25 to 86 years) with a median disease duration of nine years (IQR:4,13) and a median modified Rodnan Skin Score of 2 (IQR:0, 5). The model that best explained the impact of foot problems on QoL showed complex inter-relationships between 11 factors (Table 1). These factors explained 84 % of the impact on QoL (R^2^ = 0.84) in patients with SSc.

General disease factors, including function, breathing problems and depression had the greatest impact. Factors representing foot function and foot pain had the fourth and fifth largest effect on QoL (0.34 and 0.33 respectively): interestingly they are more influential on QoL than six other variables including gastrointestinal interference and Raynaud’s interference. A summary of the effects of the factors included in the model is provided in the Table 1.

**Conclusions**

The results from this model highlight that while systemic manifestations of SSc have a substantial impact on a person’s quality of life, foot problems are also a significant contributor to the impact on QoL; at a higher level even, than some other systemic symptoms. Clearly the importance of effective treatment of foot problems in this population is a priority, not only in addressing local foot issues, but also through their contribution to the person’s overall quality of life.

**Table 1:** Standardised direct, indirect and total effect of the variables as they relate to QoL, including foot problems

Variable	Direct effect	Indirect effect	Total effect
General function	0.454	0.186	0.640
VAS Breathing problems interference	0.000	0.493	0.493
Depression	0.270	0.158	0.428
Foot function	0.000	0.343	0.343
Foot pain	0.000	0.327	0.327
VAS Raynaud’s interference	0.000	0.294	0.294
VAS pain	0.000	0.284	0.284
Vas Disease severity	0.160	0.076	0.236
Anxiety	0.236	−0.003	0.233
VAS DU interference	0.000	0.162	0.162
VAS GI problems interference	0.000	0.095	0.095

VAS: visual analogue scale; DU: digital ulcers; GI: gastro intestinal

